# Comparison of the Keratometric Corneal Astigmatic Power after Phacoemulsification: Clear Temporal Corneal Incision versus Superior Scleral Tunnel Incision

**DOI:** 10.1155/2009/210621

**Published:** 2010-01-05

**Authors:** Yongqi He, Siquan Zhu, Ming Chen, Dejiao Li

**Affiliations:** ^1^Department of Opthalmology, Beijing Tongren Hospital, Capital Medical University, #1 Ton Giau Min alley, East district, Beijing 100730, China; ^2^Department of Opthalmology, Beijing Tongren Hospital, #1 Ton Giau Min alley, East district, Beijing 100730, China; ^3^Department of Surgery, University of Hawaii, Honolulu, Hawaii 96813-2421, USA; ^4^Department of Opthalmology, China-Japan Friendship Hospital, Ing Hwa Yuen, Zhao Yang district, Beijing 100029, China

## Abstract

*Objective*. This is prospective randomized control trial to compare the mean keratometric corneal astigmatism diopter power (not surgical induced astigmatism) among preop and one-month and three-month postop phacoemulcification of either a clear temporal corneal incision or a superior scleral tunnel Incision, using only keratometric astigmatic power reading to evaluate the difference between the two cataract surgery incisions. *Methods*. 120 patients (134 eyes) underwent phacoemulcification were randomly assigned to two groups: Group A, the clear temporal corneal incision group, and Group B, the superior scleral tunnel incision group. SPSS11.5 Software was used for statistical analysis to compare the postsurgical changes of cornea astigmatism on keratometry. *Results*. The changes of corneal astigmatic diopter in Groups A and B after 3 month postop from keratometric reading were 1.04 + 0.76 and 0.94 + 0.27, respectively (*P* = .84 >.05), which showed no statistic significance difference. *Conclusion*. The incision through either temporal clear cornea or superior scleral tunnel in phacoemulcification shows no statistic difference in astigmatism change on keratometry 3-month postop.

## 1. Introduction

 Along with the availability of the foldable intraocular lens, the incision in the phacoemulsification cataract extraction has developed from sclera incision to the clear corneal incision. At present time, cataract surgeries by phacoemulsification through clear corneal incision have become the principal method for cataract surgery because of its bloodless and fast approach. The postop SIA (surgical induced astigmatism) has always been a concern to most of surgeons. It has always been assumed that scleral incision would minimize the postop astigmatism. There were numerous studies about SIA. Masket has advocated scleral pocket with suture under tonometric and keratometric control to reduce SIA [1]. Altan-Yaycioglu et al. indicated in their study that temporal and superotemporal incisions result in only small astigmatic changes [2]. 

Conversely, the superior, superonasal, and nasal incision induced more astigmatism [2]. The review of Amesbury showed that in phacoemulsification, the incision placed on the steep corneal axis can correct small amount of astigmatism depending upon the location of the axis [3]. The peripheral corneal relaxing incision corrected greater amount of astigmatism. The toric intraocular lenses are also safe and effective for treating more than 1 diopter of astigmatism [4]. We know that the temporal clear corneal incision is efficient and bloodless whereas the superior scleral incision is less efficient and bloody. This study aims to find out that the temporal clear corneal incision will not create more SIA than superior scleral incision in three months using keratometry. Many studies on surgical induced cornea astigmatism using vector analysis of complicated Holladay-Cravy-Koch formula [5]. Since this study was focused solely on the comparison of the mean keratometric reading preop and postop in clear temporal versus superior scleral incision, the Holladay-Cravy-Koch formula regarding specific SIA was not used. Furthermore, the statistics study of ANOVA needs to pass the *F*-test for homogeneity purpose; the method of preop and postop measurement should be the same. 

 A total of 120 (134 eyes) patients were included in this study from July 2006 to January 2007 at the Ophthalmology Center, Beijing Tongren Hospital. The keratometric astigmatic diopter before and after surgery of the two groups was recorded and inputted into SPSS for statistic analysis.

## 2. Materials and Methods

### 2.1. General Data

120 patients of 134 eyes had cataract surgery at the Ophthalmology Center, Beijing Tongren Hospital, from July 2006 to January 2007, 50 male and 70 female patients. Age varied between 25 to 70 years old. There were 120 eyes suffered from senile cataract and 14 eyes had complicated cataract.

### 2.2. Methods and Grouping

The preop keratometry was done and astigmatism was recorded for all the sample patients. They were then randomly assigned to Group A or B for either the phacoemulsification through temporal clear corneal incision or the phacoemulsification through superior scleral tunnel incision with intraocular lens implant (one surgeon). It was not possible to blind the participants, staff, and the surgeon due to the natural of the intervention. However, the study personnel who verified and recorded the outcomes of keratometric astigmatism (same keratometry) was blind to the group allocation (randomly allocated). Both groups were followed and data collected in the same way. In Group A (the temporal clear corneal incision group of 70 eyes), under the topical anesthesia, the anterior chamber was entered through temporal clear cornea about 0.5 mm from the limbus with a 3.2 mm keratome. In Group B (the superior scleral tunnel incision group of 64 eyes), the scleral tunnel incision was made 2 mm behind the posterior limbus superiorly which extended 2 mm into clear cornea; anterior chamber was entered with a 3.2 mm keratome. Neither group A nor group B needs stitch to close the wound. The keratometric mean astigmatism in the two groups before surgery was similar (0.89 ± 0.42/0.91 ± 0.38). All 120 patients were able to be followed in 3 months.

### 2.3. Statistic

The comparison of the keratometric astigmatic diopter between Group A and Group B before surgery, 1-month postop, and 3-month postop was conducted using ANOVA.

## 3. Results

**Comparison of Mean between the Two Groups as Shown in Table 1**

No significance was found in the intragroup comparison of astigmatic diopter before surgery (*P* = .45 > .05). This was the test for homogeneity of variance to assess whether ANOVA was valid. Since the *P*-value was not significant (*P* = .45 > .05), this ANOVA was valid. There was a statistically difference (at 5% level) that the mean keratometric cornea astigmatic diopter in Group A was higher than that in Group B (*P* = .039 < .05) one month after surgery. However, no statistically significant difference was found when we compared the mean keratometric cornea astigmatic diopter between Group A and Group B, 3 months after surgery (*P* = .084 > .05).

The mean keratometric astigmatic diopter of Group A increased 1 month after surgery but recovered to the presurgical level 3 months after surgery. There was no statistically difference when we compared the mean keratometric corneal astigmatic diopter between postsurgical 1 month and postsurgical 3 months in Group B. The superior scleral incision showed statistically stable mean astigmatism one-month to three-month postop.

## 4. Discussion

 The clear temporal corneal incision did show statistically significant changes of more keratometric astigmatism 1 month after phacoemulsification compared to superior scleral incision. However, there was no statistically significant change after three months. It is possible that a small incision on the cornea may take up to three months to be refractive stable. In addition, the attempt of correcting astigmatism such as wound incision or limbal relaxation incision (LRI) through temporal cornea approach may get minimum effectiveness after three months. According to Holladay's study, the least SIRC (surgical induced refractive change) happened between 60 to 365 days postop [5]. 

Further study beyond 365 days in the same comparison may be necessary.

This study just intends to do the comparison between the two incisions using keratometry as a measurement as previous similar published studies [6]. 

Therefore, the axis and vector analysis was not account for during the study.

## 5. Conclusion

 Clear temporal corneal incision in phacoemulsification will not increase more keratometric corneal astigmatism than superior scleral incision after three months of operation.

## Figures and Tables

**Table 1:** Intergroup and intragroup comparison of mean keratometric cornea astigmatic diopter between Group A and Group B before and after surgery.

Group	Mean keratometric corneal (Astigmatic diopter)		
	Before surgery	1-month postop	3-month postop
A	0.89 ± 0.42	1.56 ± 0.94	1.04 ± 0.76
B	0.91 ± 0.38	1.09 ± 1.03	0.94 ± 0.27
